# Tailoring Photoluminescence from Si-Based Nanocrystals Prepared by Pulsed Laser Ablation in He-N_2_ Gas Mixtures

**DOI:** 10.3390/molecules25030440

**Published:** 2020-01-21

**Authors:** Anastasiya A. Fronya, Sergey V. Antonenko, Alexander Yu. Kharin, Andrei V. Muratov, Yury A. Aleschenko, Sergey I. Derzhavin, Nikita V. Karpov, Yaroslava I. Dombrovska, Alexander A. Garmash, Nikolay I. Kargin, Sergey M. Klimentov, Victor Yu. Timoshenko, Andrei V. Kabashin

**Affiliations:** 1 MEPHI, Institute of Engineering Physics for Biomedicine (PhysBio), Kashirskoe sh. 31, 115409 Moscow, Russia; AAFronya@mephi.ru (A.A.F.); SVAntonenko@mephi.ru (S.V.A.); AYKharin@mephi.ru (A.Y.K.); AYAleschenko@mephi.ru (Y.A.A.); sderzhavin@kapella.gpi.ru (S.I.D.); NVKarpov@mephi.ru (N.V.K.); yaroslava.dombrovska@gmail.com (Y.I.D.); AAGarmash@mephi.ru (A.A.G.);; 2 Lebedev Physical Institute of the Russian Acad. Sci., Leninskiy Pr. 53, 119991 Moscow, Russia; muratov@lebedev.ru; 3 MEPHI, Institute of Nanoengineering in Electronics, Spintronics and Photonics, Kashirskoe sh. 31, 115409 Moscow, Russia; NIKargin@mephi.ru; 4 Prokhorov General Physics Institute of the Russian Acad. Sci., Vavilova St. 38, 117942 Moscow, Russia; 5 Lomonosov Moscow State University, Physics Dep., Leninskie Gory 1, 119991 Moscow, Russia; 6 Aix Marseille Univ, CNRS, LP3, Campus de Luminy, Case 917, 13288 Marseille, France

**Keywords:** pulsed laser ablation in gases, pulsed laser deposition, silicon nanoparticles, silicon quantum dots, silicon oxynitride, photoluminescence, quantum confinement, bioimaging

## Abstract

Using methods of pulsed laser ablation from a silicon target in helium (He)-nitrogen (N_2_) gas mixtures maintained at reduced pressures (0.5–5 Torr), we fabricated substrate-supported silicon (Si) nanocrystal-based films exhibiting a strong photoluminescence (PL) emission, which depended on the He/N_2_ ratio. We show that, in the case of ablation in pure He gas, Si nanocrystals exhibit PL bands centered in the “red - near infrared” (maximum at 760 nm) and “green” (centered at 550 nm) spectral regions, which can be attributed to quantum-confined excitonic states in small Si nanocrystals and to local electronic states in amorphous silicon suboxide (a-SiO_x_) coating, respectively, while the addition of N_2_ leads to the generation of an intense “green-yellow” PL band centered at 580 nm. The origin of the latter band is attributed to a radiative recombination in amorphous oxynitride (a-SiN_x_O_y_) coating of Si nanocrystals. PL transients of Si nanocrystals with SiO_x_ and a-SiN_x_O_y_ coatings demonstrate nonexponential decays in the micro- and submicrosecond time scales with rates depending on nitrogen content in the mixture. After milling by ultrasound and dispersing in water, Si nanocrystals can be used as efficient non-toxic markers for bioimaging, while the observed spectral tailoring effect makes possible an adjustment of the PL emission of such markers to a concrete bioimaging task.

## 1. Introduction

Nanostructured silicon (Si) has attracted a lot of attention for the last couple of decades due to a series of unique properties opening up avenues for diverse applications [1,2,3]. Biomedicine looks to be one of main beneficiaries of these properties, as Si nanoparticles (NPs) are not only highly biocompatible [4] and biodegradable [5,6], but can also serve as contrast agents in bioimaging [7,8,9], sensitizers of various therapies [10,11,12,13] and carriers of anticancer drugs [5] and radionuclides [14].

The bioimaging functionality of Si-NPs typically relies on photoluminescence (PL) of quantum-confined excitons in Si nanocrystals and/or defect-related states [4,15] that makes possible an efficient light emission in the region of relative transparency of biotissues (600–850 nm). To enable highly emissive Si-based quantum dots (QDs), one has to form high quality nanocrystals and properly passivate them in order to remove non-radiative centers [16,17]. Porous silicon technology based on electrochemical etching of crystalline Si (c-Si) wafers in hydrofluoric acid solutions presents a straightforward way to form such QDs, while a hydroxyl-based passivation of the nanocrystals offered by the combination of hydrogenation and oxidation (during synthesis and subsequent storage in air, respectively) ensures a good dispersion of Si-QDs in aqueous solutions [2,4,5]. However, porous silicon structures can be contaminated by acid derivatives [18], which complicates biological prospects of such QDs. Alternative “dry” fabrication methods can offer much better purity of Si nanocrystals [7,19,20], but in many cases one has to apply a similar wet chemistry-based etching step in acid solutions to enable the PL emission and disperse Si-QDs in water [7,19].

Si nanocrystals synthesized by methods of laser ablation present a viable alternative to nanostructures prepared by conventional chemical methods to offer required non-toxicity option for biomedical applications [21,22]. Such methods profit from a natural production of nanoclusters during the interaction of pulsed laser radiation with a target, and their subsequent growth inside a partially ionized ionized plasma plume [23,24,25]. When ablated in a liquid ambient, the nanoclusters can be grown in a controllable manner to obtain solutions of colloidal NPs [26,27,28], which can be visualized by non-linear responses [29] and used as sensitizers in various therapies, including photodynamic therapy [26] and radiofrequency radiation-induced [12] hyperthermia. When ablated in gaseous ambience, the nanoclusters can be deposited on the target itself [30,31] or on a separate substrate [32,33,34] to form a nanocrystalline porous film exhibiting strong PL emission. In both cases, the properties of laser-ablated nanostructures can differ from counterparts prepared by conventional methods and demonstrate a great potential for energy and healthcare applications [22].

We recently developed a simple methodology based on pulsed laser deposition (PLD) in residual helium gas to fabricate Si-based nanocrystalline films, which exhibit bright (quantum yield >5%) PL around 750–850 nm due to quantum confinement of exciton carriers [9]. It is important that the generation of such emission band does not require an additional wet chemistry-based treatment step in hydrofluoric acid solutions, as it usually takes place in the case alternative “dry” fabrication techniques such as laser pyrolysis of silane [9,17]. We also demonstrated the possibility of the preparation of quantum dots for bioimaging on the basis of the as formed Si nanocrystals. To do this, the nanocrystals were removed from the substrate by ultrasound and dispersed in aqueous solutions. Being tested as markers in bioimaging in cellular models, the Si-QDs demonstrated high efficiency in providing optical contrast in the absence of toxic effects [9]. The proposed PLD-based nanocrystal synthesis methodology, followed by nanocrystal milling and water-dispersion, looks as a promising way to prepare non-toxic, biodegradable QDs for bioimaging. However, the emission of these QDs is fixed in the red-infrared range, which limits their application area. In addition, the observed PL still needs enhancement to improve contrast of images.

Here, we solve the tuneability problem of emission spectra from nanocrystals synthesized by the PLD method. We demonstrate the possibility for tailoring PL emissions toward a green-yellow range under a drastic increase of its intensity by the addition of nitrogen gas to buffer helium gas during the synthesis procedure. The PL tailoring effect was explained by a nitrogen-based passivation of Si nanocrystals in order to enable appropriate radiative centers. It is expected that such a tailoring effect will help to match different spectral regions according to a concrete bioimaging task.

## 2. Results and Discussion

**Figure 1:**
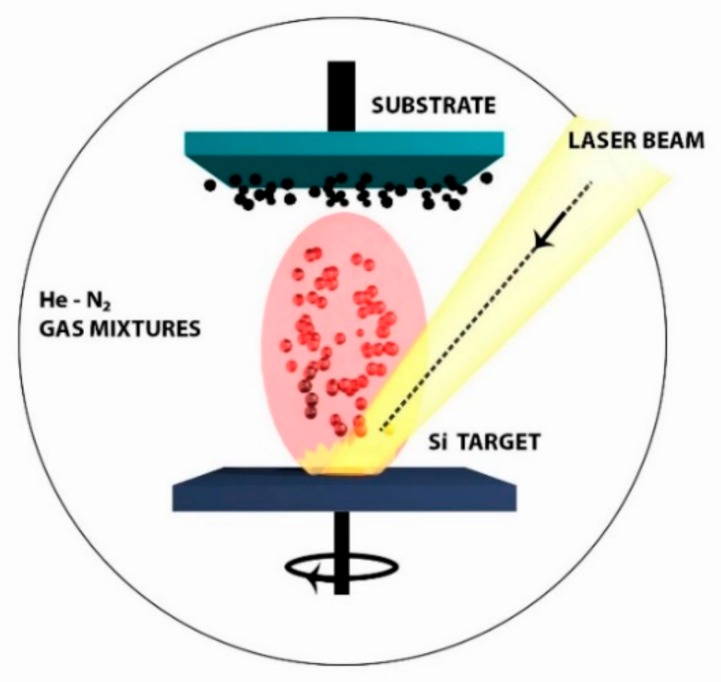
Scheme of pulsed laser ablation to deposit Si-based nanostructured films. A beam from UV excimer laser is directed at the angle of 45 deg. onto a rotated Si target. The ablated material is deposited on a substrate located 2–3 cm above the target surface. The target–substrate distance is selected in such a way that visible plasma plume area contacts the substrate surface.

Our experiments were carried out using a methodology described in the Section 3. Briefly, a focused beam from a KrF excimer laser (248 nm) was used to irradiate a c-Si target at the angle of 45 deg. in the presence of He-N_2_ gas mixtures maintained at reduced pressures of 0.5–5 Torr (Figure 1). The target was constantly rotated in order to minimize the ablation of material from the same place on the target surface. A laser-initiated plasma plume expanded perpendicularly to the target surface and could be observed by a characteristic green emission associated with the PL of Si clusters in the vapor phase [23]. In this case, the presence of a buffer gas at reduced pressure enables one to cool down ablated nanoclusters in order to control the Si condensation and crystallization processes [33,34]. The nanoclusters were then deposited on a substrate (c-Si wafer or glass slide) placed at some distance from the target, as shown in Figure 1. Such a deposition process led to the formation of Si-based nanostructured film after several thousands of laser pulses. The ablation was done under different proportions of N_2_ and He in gas mixtures R = P_N2_/P_He_. Table 1 presents parameters of the three most representative samples.

**Figure 2:**
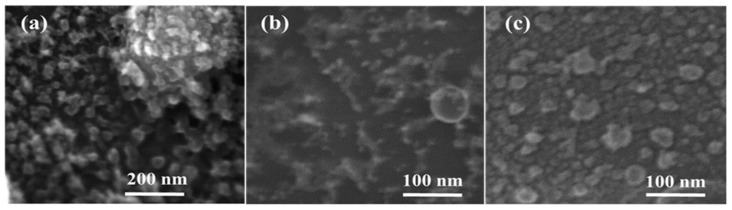
Top-view SEM images of laser-ablated samples obtained at different ratio R between partial pressures of helium and nitrogen: R = 0 (**a**); R = 0.1 (**b**); R = 1 (**c**).

Scanning electron microscopy (SEM) showed that all deposited films exhibited nanograin morphology (Figure 2). Our previous studies of the ablation in pure He atmosphere showed that the prepared films were composed from 2–4 nm Si nanocrystals embedded in amorphous silicon suboxide (SiO_x_, 0 < × <1) matrix [34]. The films deposited in pure He and in He-N_2_ mixtures could have slightly different structural morphologies: the nanocrystals combined together to form larger 20–30 nm nanograin aggregates in the case of pure He (Si-1) (a), while in the case of He-N_2_ mixtures (Si-2, Si-3), such nanograins were below 10 nm (b,c). Such a difference in the morphologies of Si-NPs can be explained by the fact that N_2_ molecules are much heavier than He atoms, which led to a faster condensation of Si nanoclusters under their collisions with gas species and subsequent earlier crystallization before reaching the substrate to form smaller NPs.

Transmission electron microscopy (TEM) studies confirmed a nanocrystalline structure of samples prepared in both pure He atmosphere and He/N_2_ mixtures (Supplementary Figures S1–S3). Although electron diffraction patterns indicated certain contribution of the amorphous phase in films deposited in the He:N_2_ mixtures (Supplementary Figure S3b), they were still predominantly crystalline. X-ray diffraction (XRD) also confirmed the nanocrystalline structure of all prepared samples (Supplementary Figure S4). Mean nanocrystal size estimated from XRD peaks using Debye-Scherrer equation was in the range of 30–40 nm and showed a tendency of slight decrease while partial nitrogen pressure was increased (Supplementary Figure S5). Note that the used estimation typically provides the largest size of crystals in an ensemble.

**Figure 3:**
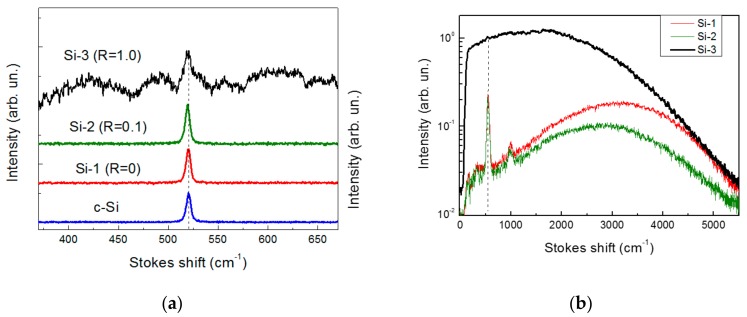
(**a**) Raman spectra of nc-Si sample obtained at different ratio of helium to nitrogen pressures, as well as one for c-Si substrate; (**b**) Raman and PL spectra measured in a wide range. Vertical dashed lines indicate the Raman line position (520.5 cm^−1^) for c-Si.

The presence of Si nanocrystals in PLD-prepared films was confirmed by the results of the Raman spectroscopy, which is known as a standard tool to probe the size and crystallinity of Si-based nanomaterials [35]. Raman shifts and spectral broadening arising from effects of phonon confinement in Si-nanocrystals are in fact fingerprints of nanocrystals having sizes between 1 and 10 nm [35]. A quantitative analysis of Raman spectra allows one to assess both the mean nanocrystal size and the ratio between the crystalline and amorphous phases in Si-based nanostructures [36]. Figure 3a shows Raman spectra of the samples deposited at different nitrogen/helium ratios and that of a c-Si substrate for comparison. It is visible that the Raman spectra of samples Si-1 (R = 0) and Si-2 (R = 0.1) exhibited a sharp line centered at 519.0 and 519.5 cm^−1^, respectively, that was close to a Raman line of c-Si substrate at 520.5 cm^−1^. A slight low-energy shift of the Raman lines for these samples gives evidence for the production of Si-QDs with sizes below 10 nm [35]. The Raman spectra from sample Si-3 contained the same lines and three additional broad lines around 400–450, 480–490, and 600–620 cm^−1^. While 480–490 cm^−1^ line could be attributed to amorphous Si [36], the bands at 400–450 and 600–620 cm^-1^ looked close to the S-N vibration frequencies [37,38]. Note that the observed high level of noise for the Raman spectrum for Si-3 sample could be explained by the PL background. Indeed, the same spectra recorded in a wide spectral range revealed a broad PL band, whose intensity was much stronger in the case of Si-3 sample (Figure 3b).

**Figure 4:**
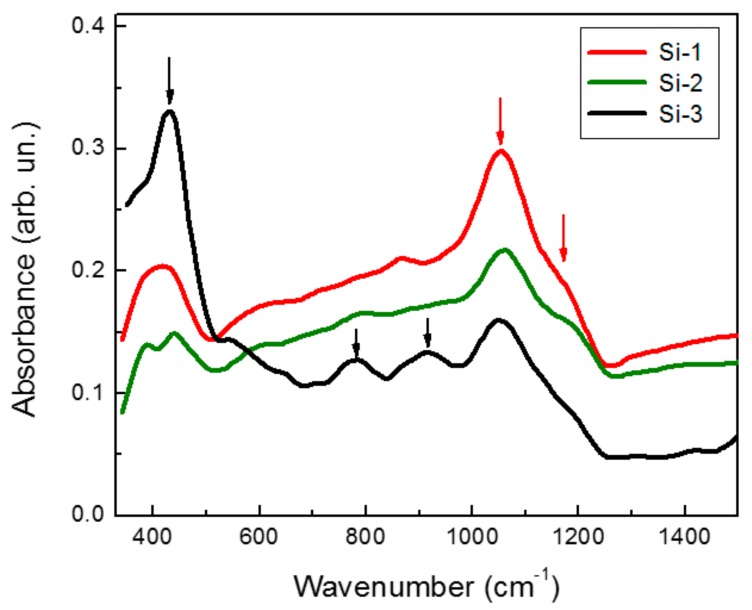
Fourier transform infrared (FTIR) absorbtion spectra of laser-ablated samples obtained at different ratio of helium to nitrogen pressures. Vertical red and black arrows indicate the spectral position of the Si-O and Si-N bonds, respectively.

Figure 4 shows FTIR-ATR spectra of the samples. It is reasonable to assume that the deposition of films in nitrogen gas environment (Si-2, Si-3) could lead to the formation of silicon nitride (Si-N) bonds. Indeed, in addition to a strong absorption peak at about 1050 cm^−1^ with shoulder at 1070 nm related to the O-Si-O bonds, three peaks at about 450, 800, and 900 cm^−1^ appeared in the spectrum of the sample deposited at higher partial pressure of nitrogen (Si-3). The latter peaks could be associated with the S-N vibration in amorphous silicon oxynitride, i.e., a-SiN_x_O_y_ [39,40,41]. Note that spectral positions of S-N bonds slightly differed from relevant values in pure silicon nitride (Si_3_N_4_) due to a certain oxygen content in a-SiN_x_O_y_ films, which was probably appeared in the coating of Si nanocrystals during their aging in air [40,42].

**Figure 5:**
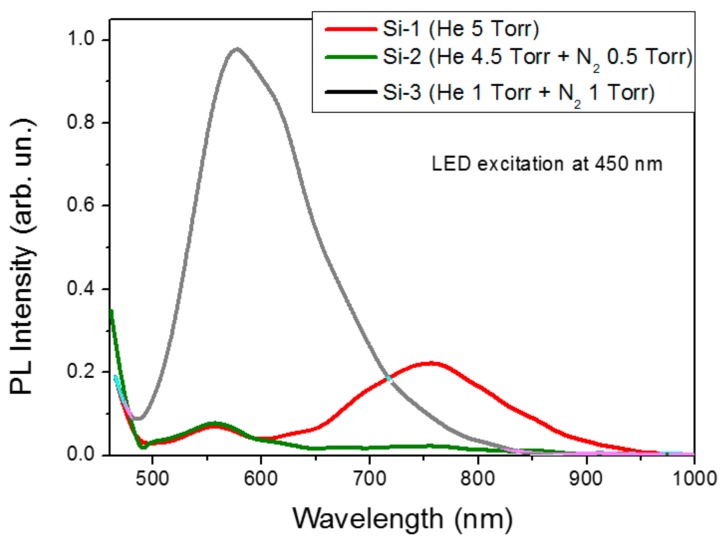
Photoluminescence spectra of laser-ablated Si films prepared under different gas mixtures: 5 Torr He (red); 4.5 Torr He + 0.5 Torr N_2_ (green); 1 Torr He + 1 Torr N_2_ (black).

Just after their exposition to ambient air, laser-ablated films exhibited strong PL signals under excitation at 450 nm, as shown in Figure 5. The PL spectrum for the films deposited in pure He (sample Si-1, red line) consists of two PL bands centered around 760 nm and 550 nm, respectively, which is consistent with results previous studies [9,33,34]. As we showed in Ref. [9], the first “red” band is due to the radiative exciton transitions in Si-QDs, while the “green” band can be explained by the radiative transitions via the localized electronic states in silicon suboxide coating of Si-QDs under the aging of laser-ablated films in ambient air [9,34]. As follows from the spectrum for Si-2 sample (green line), the addition of a small (10%) amount of N_2_ leads to immediate quenching of the exciton PL band, while the green band remains unaffected. However, a further increase of N_2_ content (sample Si-3, black line) leads to the evolution of the second band into at least 10-times more intense band in the yellow range (center at 570–580 nm). The generation of this green-yellow band can be unambiguously related to nitrogen-based passivation of Si-QDs and attributed to the radiative transitions between electronic states in a-SiN_x_O_y_ coating on the surface of Si nanocrystals [43,44].

**Figure 6:**
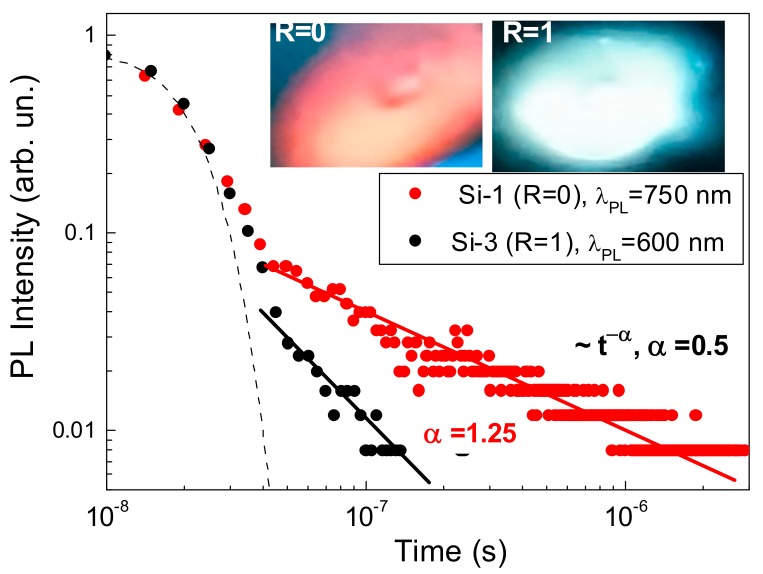
PL transients from samples Si-1 and Si-3 obtained for two different ratios between helium and nitrogen pressures (R = 0 and R = 1, respectively). Dashed line indicates time response of the detection system, while solid black and red lines are power law approximations with exponents of 0.5 and 1.25, respectively. Left and right insets show digital images of the PL spots (2 × 3 mm^2^) on the top of samples Si-1 and Si-3, respectively. PL was excited by 20 ns laser pulses at 351 nm.

Such a supposition on the origin of “red” and “green-yellow” PL bands was confirmed by measurements of PL transients under the excitation by 20 ns laser (wavelength 351 nm). As shown in Figure 6, the samples obtained in pure He and He-N_2_ mixtures exhibited non-exponential PL decays with rates depending on the nitrogen content in the mixture. The sample deposited in pure He-based ambient (Si-1, red line) demonstrated PL transients according to the power law with an exponent of about 0.5 that agrees with results of our previous studies [9,45]. Such a slow descent of PL intensity evidences a pathway of the radiative recombination, which can be controlled by dissipative tunneling in a network of interconnected Si nanocrystals [45]. In contrast, the “green-yellow” PL band from Si-3 sample (black line) was characterized by faster PL transient, which could be extrapolated by a power-law decay but with an exponent of about 1.25. The faster PL decay for sample Si-3 could be explained by a higher rate of the radiative recombination between localized electronic states in a-SiN_x_O_y_ coating of Si-NCs similarly to the fast PL decay in a-SiN_x_O_y_ films grown by PECVD [37]. As a conclusion, the observed “red” band could be attributed to the confined excitonic states in Si nanocrystals-QDs, while the “green-yellow” band with higher relative intensity was related to local electronic states in a-SiN_x_O_y_ coating of Si-QDs due to specific nitrogen-based passivation.

Thus, the addition of nitrogen to helium atmosphere during pulsed laser ablation of silicon target drastically changed PL properties of the prepared Si-based nanostructured films. Indeed, instead of having a strong PL band in the spectral region of 730–780 nm, we could observe a five-fold stronger PL band in the spectral range of 550–620 nm. It is important that the generation of both bands was achieved in the absence of wet chemistry steps, which excludes the presence of any toxic substances in the formed Si nanocrystals. Such a tuning opportunity for the PL properties looks very promising for projected bioimaging applications. As it was showed in our previous study [9], laser-ablated Si nanocrystal-based films could be easily milled by the ultrasound and dispersed in aqueous solutions, including physiological solutions, to form Si-based luminescent NPs. In this case, exciton-based band could be used to track the presence of these QDs in different cell compartments. The samples prepared in He-N_2_ atmosphere can further enrich the imaging ability of such Si-NPs. First, the observed “green-yellow” band appears to be five-times stronger compared to the exciton-based “red” PL. In addition, the observed high-energy shift of PL spectrum looks favorable for some bioimaging applications, taking into account that the “green yellow” band is highly efficient (see the right inset in Figure 6) and is still partially in the window of relative tissue transparency.

We suppose that the ultrapure Si-based NPs prepared from laser-synthesized nanostructured Si layers can also bring novel therapy modalities, including the photodynamic therapy [10], RF-induced hyperthermia [12], infrared laser-induced hyperthermia [11]. Here, the combination of these therapy functionalities with the imaging option based on the observed green, yellow and red PL bands promises the development of new theranostic agents combining the therapy and diagnostic functionality in one entity.

## 3. Materials and Methods

### 3.1. Materials

Single-crystal (100)-oriented Si wafers (optically polished, with a diameter of 100 mm, p-type conductivity, specific electrical resistivity 10 Ohm·cm, thickness 0.4 mm) were used as targets in laser-ablative experiments. The same wafers were used as substrates for the deposition of Si-based nanostructured films.

### 3.2. Pulsed Laser Ablation for the Deposition of Si-Based Nanostructured Films

The target was ablated using a fully automated pulsed laser deposition system (MBE-2000, PVD Products, Wilmington, MA, USA), which was based on a krypton fluoride excimer laser COMPexPro with wavelength 248 nm, laser pulse length 30 ns, repetition rate from 1 to 105 Hz. The use of ultraviolet laser radiation from excimer lasers in PLD schemes is justified by a strong absorption of this radiation by a largest majority of materials and its transparency for formed plasma plume [23,32,33,34]. The output laser energy was varied in the interval of 50–300 mJ, corresponding to the average output power of 1–25 W. The energy value during the deposition process was maintained constant (deviations less than 10%).

Pulsed laser ablation experiments were carried out in a chamber, which was initially pumped out to a residual pressure of 10^−7^ Torr. Then the chamber was filled with buffer gases (He, N_2_) to fix the operation pressure in the range of 0.5–5 Torr. In our experiments, the target was constantly rotated and irradiated at the incident angle of 45 deg. to initiate ablation of material perpendicularly to the target surface. The material was then deposited on a substrate placed at 2 cm from the target. Such a distance from was selected as optimum for a given laser pulse energy and range of operation pressures. The pulsed laser ablation process resulted in the formation of Si-based nanostructured films having the thickness of about 1 μm after 5000–7000 of laser pulses. Table 1 presents the parameters of three most representative samples.

### 3.3. Characterization of Nanoparticles

SEM system (TESCAN MAIA 3) operating at 0.1–30 kV was used to study the morphology of laser-ablated films. TEM analyses was carried out by using a LEO912 AB OMEGA transmission electron microscope. The composition and crystallinity of the films was studied by means of the Fourier-transform infrared spectroscopy (FTIR) in attenuated total reflectance (ATR) mode and Raman spectroscopy, respectively. The FTIR-ATR measurements were carried out by using a Tensor 27 FTIR spectrometer (Bruker Optik GmbH, Ettlingen, Germany) with an MVP-Pro Star single reflection diamond ATR accessory. The Raman measurements were done by using a confocal micro-Raman spectrometer (Confotec MR350, SOL Instr.) under laser excitation at 632.8 nm. During the Raman diagnostics a special attention was paid to avoid overheating of the samples under focused laser excitation. PL was excited either by continuous wave radiation of a “blue” LED (Guangzhou Mingnuo Electronics Co., Ltd., Guangzhou, China) at 450 nm with intensity of 10 mW/cm^2^ or by a nanosecond laser (TECH-35 Basic, Laser-export Co., Ltd., Moscow, Russia) with pulse duration of 20 ns, wavelength 351 nm, energy density per pulse 100 nJ/cm^2^, repetition rate 1 kHz. PL spectra and transients were detected by using a Mightex HRS CCD-spectrometer (Mightex Systems, Toronto, ON, Canada) and a grating monochromator equipped with a photomultiplier, respectively. Transients of the PL signal and photocurrent were measured by using a 500 MHz digital oscilloscope with a time resolution of about 1 ns.

## 4. Conclusions

We used methods of pulsed laser ablation in residual gaseous ambient (0.5–5 Torr) to fabricate Si-based nanostructured films on a substrate. We show that in the case of ablation in pure He gas Si nanocrystals exhibit PL centered in near-infrared (maximum at 760 nm) and green (550 nm) range, while the addition of N_2_ leads to the quenching of infrared band and the generation of five-times more intense green-yellow PL band centered at 580 nm. The generation of the latter band was explained by the formation of localized radiative centers in a-SiN_x_O_y_ coating of Si nanocrystals, which was confirmed by measurements of the PL transient. The prepared laser-synthesized Si nanocrystals can be used as markers for bioimaging, while tuned PL emission makes possible the adjustment of such markers to a concrete bioimaging task.

## Figures and Tables

**Table 1:** Representative samples of nanocrystalline silicon prepared at different partial pressures of helium (P_He_) and nitrogen (P_N2_).

Notation of Sample	P_He_, Torr	P_N2_, Torr	R = P_N2_/P_He_
Si-1	5.0	0	0
Si-2	5.0	0.5	0.1
Si-3	1.0	1.0	1.0

## Supplementary Materials

The following are available online at https://www.mdpi.com/1420-3049/25/3/440/s1, Figure S1: TEM images (a) and electron diffraction pattern (b) of NPs from S-1 sample, Figure S2: TEM images (a) and electron diffraction pattern (b) of NPs from S-2 sample, Figure S3: TEM images (a) and electron diffraction pattern (b) of NPs from S-3 sample, Figure S4: XRD spectra of NPs from samples S-1, S-2 and S-3, Figure S5: XRD spectra and their analysis in a vicinity of the (111) peak for NPs from samples S-1 (a), S-2 (b), S-3 (c).
