# Supplementary material for: Tailoring Photoluminescence from Si-Based Nanocrystals Prepared by Pulsed Laser Ablation in He-N2 Gas Mixtures
Source: Molecules. 2020 Jan 21;25(3):440. doi: 10.3390/molecules25030440 (PMC7037818; doi:10.3390/molecules25030440)
Supplement: Supplementary file 1 [file molecules-25-00440-s001.pdf]

Supplementary Materials to

Tailoring photoluminescence from Si-based nanocrystals prepared by pulsed laser ablation in He-N_2 gas mixtures

Anastasiya A. Fronya[1,2], Sergey V. Antonenko[1,3], Alexander Yu. Kharin[1], Andrei V. Muratov[2], Yury A. Aleschenko[1,2], Sergey I. Derzhavin[1,4], Nikita V. Karpov[1], Yaroslava I. Dombrovska[1], Alexander A. Garmash[1,3], Nikolay I. Kargin[3], Sergey M. Klimentov[1], Victor Yu. Timoshenko[1,2,5]*, Andrei V. Kabashin[1,6]*

[1] MEPHI, Institute of Engineering Physics for Biomedicine, Kashirskoe sh. 31, 115409 Moscow, Russia
[2] Lebedev Physical Institute of the Russian Acad. Sci., Leninskiy Pr. 53, 119991 Moscow, Russia
[3] MEPHI, Institute of Nanoengineering in Electronics, Spintronics and Photonics, Kashirskoe sh. 31, 115409 Moscow, Russia
[4] Prokhorov General Physics Institute of the Russian Acad. Sci., Vavilova St. 38, 117942 Moscow, Russia
[5] Lomonosov Moscow State University, Physics Dep., Leninskie Gory 1, 119991 Moscow, Russia
[6] Aix Marseille Univ, CNRS, LP3, Campus de Luminy, Case 917, 13288 Marseille, France
* Correspondence: vtimoshe@gmail.com (V.Y.T), kabashin@lp3.univ-mrs.fr (A.V.K)

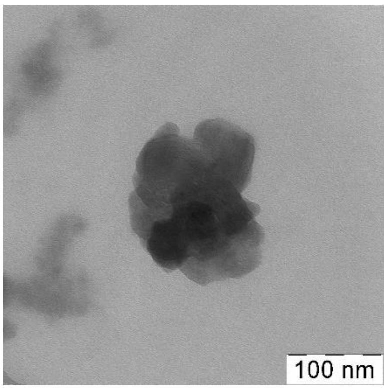

(a)

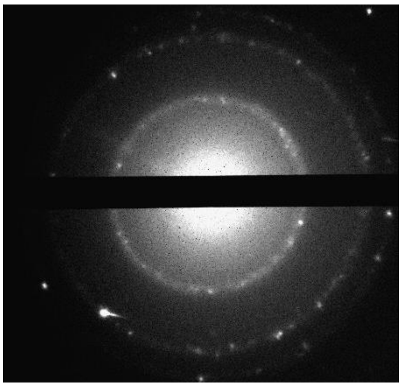

(b)

Figure S1. TEM images (a) and electron diffraction pattern (b) of NPs from S-1 sample.

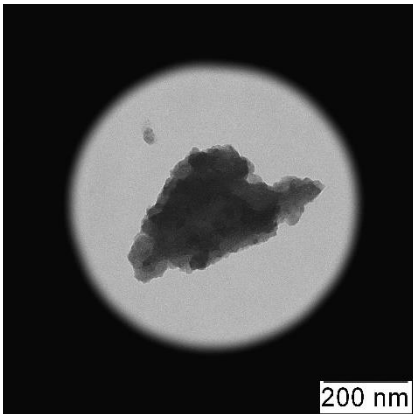

(a)

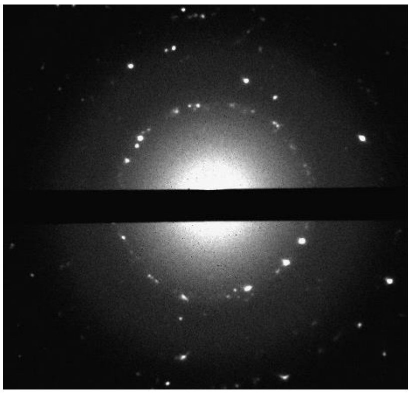

(B)

Figure S2. TEM images (a) and electron diffraction pattern (b) of NPs from S-2 sample.

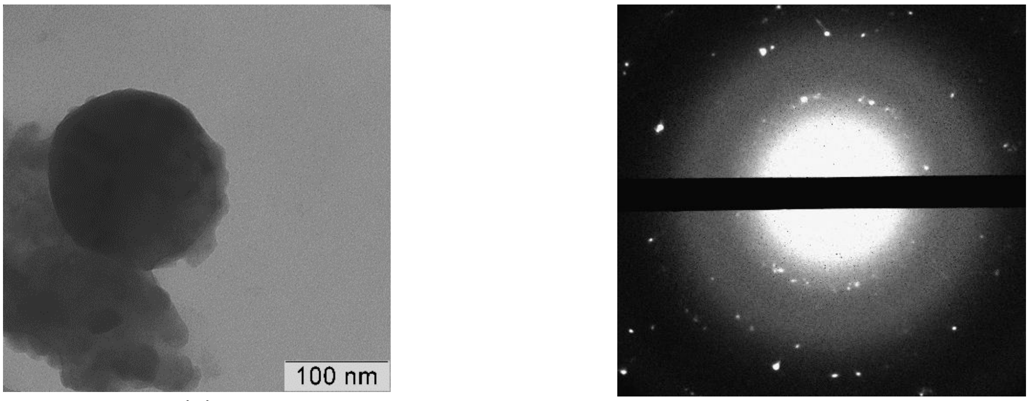

(a) (b)

Figure S3. TEM images (a) and electron diffraction pattern (b) of NPs from S-3 sample.

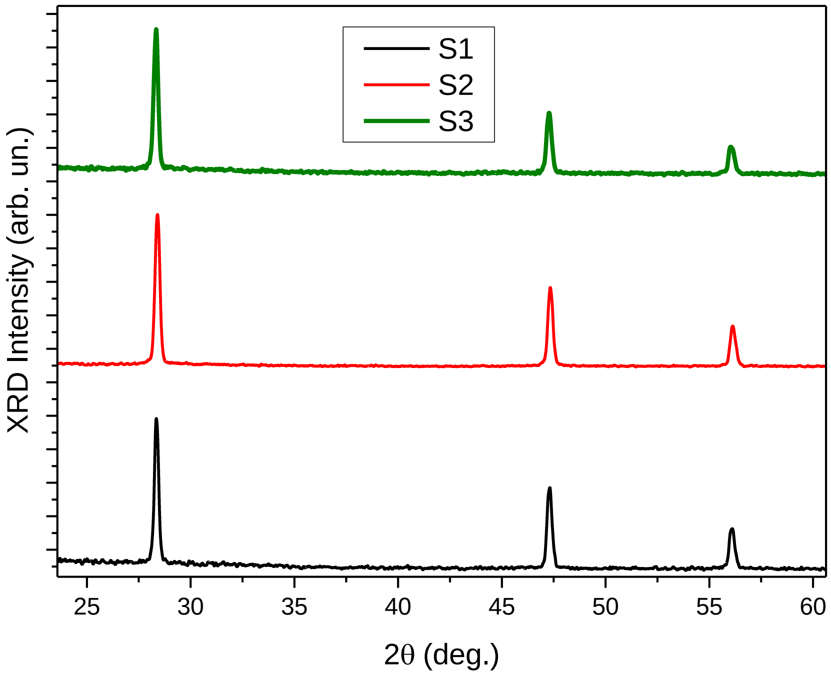

Figure S4. XRD spectra of NPs from samples S-1, S-2 and S-3.

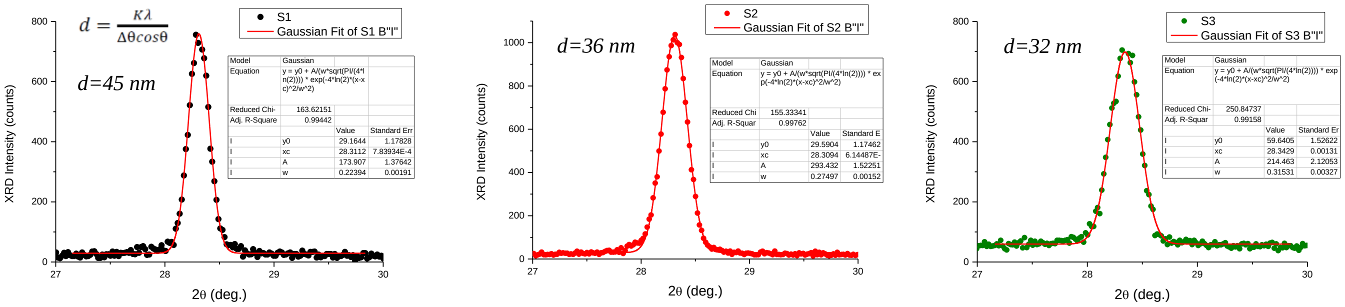

(a) (b) (c)

Figure S5. XRD spectra and their analysis in a vicinity of the (111) peak for NPs from samples S-1 (a), S-2 (b), S-3 (c).
